# Improved time to exhaustion following ingestion of the energy drink Amino Impact™

**DOI:** 10.1186/1550-2783-7-14

**Published:** 2010-04-15

**Authors:** Allyson L Walsh, Adam M Gonzalez, Nicholas A Ratamess, Jie Kang, Jay R Hoffman

**Affiliations:** 1 The College of New Jersey, Ewing, NJ 08628-0718, USA

## Abstract

**Background:**

The purpose of this study was to examine the effect of a commercially available energy drink on time to exhaustion during treadmill exercise. In addition, subjective measures of energy, focus, and fatigue were examined

**Methods:**

Fifteen subjects (9 men and 6 women; 20.9 ± 1.0 y; 172.1 ± 9.1 cm; 71.0 ± 9.4 kg; 16.9 ± 9.7% body fat) underwent two testing sessions administered in a randomized, double-blind fashion. Subjects reported to the laboratory in a 3-hr post-absorptive state and were provided either the supplement (SUP; commercially marketed as Amino Impact™) or placebo (P). During each laboratory visit subjects performed a treadmill run (70% VO_2 _max) to exhaustion. Mean VO_2 _was measured during each endurance exercise protocol. Subjects were required to complete visual analog scales for subjective measures of energy, focus and fatigue at the onset of exercise (PRE), 10-mins into their run (EX10) and immediately post-exercise (IP).

**Results:**

Time to exhaustion was significantly greater (p = 0.012) during SUP than P. Subjects consuming the supplement were able to run 12.5% longer than during the placebo treatment. Subjects consuming SUP reported significantly greater focus (p = 0.031), energy (p = 0.016), and less fatigue (p = 0.005) at PRE. Significant differences between groups were seen at EX10 for focus (p = 0.026) and energy (p = 0.004), but not fatigue (p = 0.123). No differences were seen at IP for either focus (p = 0.215), energy (p = 0.717) or fatigue (p = 0.430).

**Conclusions:**

Results of this study indicate that the supplement Amino Impact™ can significantly increase time to exhaustion during a moderate intensity endurance run and improve subjective feelings of focus, energy and fatigue.

## Introduction

The use of pre-exercise energy drinks has become a popular supplementation habit among recreational and competitive athletic populations. Recent studies have indicated that among American adolescents and young adults energy drinks are second only to multivitamins in popularity [1,2], with reports suggesting that 30% of this population group regularly consumes energy drinks [2]. Energy drinks are reported to be quite popular within athletic populations as well [1,3,4]. Petroczi and colleagues [4] reported that more than 40% of British athletes self-admitted to using energy drinks to enhance their workouts or performance. Another study indicated that 89% of athletes competing in the Ironman World Triathlon Championships admitted that they were planning on using caffeinated supplements prior to competition [3]. Athletes from across the performance spectrums (endurance athletes to strength/power athletes) consume energy drinks. However, it is not known whether one type of athlete consumes energy drinks more frequently than another. Anecdotally, it appears that certain energy drinks may be more common for the endurance athlete, while others may be marketed more to the strength/power athlete. This may be partly attributed to the widely reported benefits that caffeine, an ingredient common in energy drinks, has on endurance performance but not on anaerobic performance [5-11].

Caffeine has been shown to be an effective ergogenic agent by delaying fatigue and increasing time to exhaustion during endurance exercise [5-9]. Its efficacy as an ergogenic aid during anaerobic exercise and strength/power events though is limited [8,10,11]. Recent studies have examined energy drinks that have been marketed primarily to the strength/power athlete [12,13]. These studies investigating a pre-exercise drink comprised of caffeine in combination with taurine, glucuronolactone, and branched chain amino acids (BCAA) reported significant improvements in the volume of training (expressed as number of repetitions performed during a bout of resistance exercise) when these supplements were consumed 10 minutes prior to the training session. The greater number of repetitions performed during the training session were associated with a greater anabolic response (elevations in growth hormone) [12].

Recently, a new energy drink has been developed using ingredients similar to those previously discussed studies showing enhanced resistance exercise performance. Considering that many of the ingredients within the energy supplements marketed to the strength/power athlete are similar to that found in supplements used for the endurance athlete, it is of interest to determine whether the ergogenic benefits cross performance spectrums. Interestingly, previous studies that have shown efficacy of a specific energy supplement for one mode of exercise (e.g., endurance exercise) have failed to see similar efficacy in a different exercise protocol (e.g. resistance exercise) [8]. Thus, the purpose of this study is to examine the acute effects of a pre-exercise energy supplement using ingredients previously demonstrated to enhance resistance training performance on time to exhaustion during treadmill exercise, and on subjective feelings of focus, energy and fatigue in healthy, physically active college-aged men and women.

## Methods

### Subjects

Fifteen recreationally active subjects (9 men and 6 women; 20.9 ± 1.0 y; 172.1 ± 9.1 cm; 71.0 ± 9.4 kg; 16.9 ± 9.7% body fat) underwent two testing sessions administered in a randomized and double-blind fashion. Subjects were recruited from The College of New Jersey through announcements in the Health and Exercise Science Department. Following an explanation of all procedures, risks, and benefits associated with the experimental protocol, each subject gave his/her written consent prior to participating in this study and completed a medical history/physical activity questionnaire to determine eligibility. Subjects who were pregnant, smokers, taking any medication, had any known metabolic or cardiovascular disease, and/or psychiatric disorder were excluded from the study. Subjects were also required to have been free of any nutritional supplements or ergogenic aids for 6 weeks preceding the study, and were asked to refrain from taking any additional supplement(s) during the course of the study.

### Study Design

The study followed a double-blind, crossover design. Subjects reported to the Human Performance Laboratory on three separate occasions. During the first session subjects performed a maximum oxygen consumption (VO_2_max) test. During the subsequent two testing session's subjects performed the experimental trials at a standardized time of day. Each testing session was separated by approximately one week (8.4 ± 2.2 days). Subjects were instructed to refrain from consuming any caffeine products on the day of each testing session and from performing any strenuous physical activity for the previous 12 hours. In addition, subjects were instructed to be at least 3 hours post-absorptive state prior to each trial. During each visit to the laboratory subjects were seated for 10 min. Following this resting period subjects were randomly provided either the energy drink supplement (SUP) or a placebo (P). The supplement was provided according to the manufacturer's serving recommendation (26 g of Amino Impact™ mixed in 500 ml of water). On the subject's second visit to the laboratory they were provided with the opposite treatment.

### VO_2_max Test

The VO_2_max test was conducted on a treadmill (Desmo model, Woodway^®^, Waukesha, WI) and followed an incremental testing protocol. Briefly, this protocol required the subject to begin exercise at a self-selected speed between 134 and 188 m·min^-1^. For the duration of the test, the self-selected speed was maintained while the treadmill elevation increased by 2% every 2 minutes. The test was preceded by a 5 min warm-up (self-selected running speed at 0% grade), and was terminated at volitional exhaustion. Immediately following the warm-up period subjects were fitted with a Medgraphics preVent™ pneumotach (Medical Graphics Corporation, St. Paul, MN) to measure oxygen uptake (VO_2_) and respiratory exchange ratio (RER) through open-circuit spirometry using a metabolic measurement cart (CPX Ultima™ series, Medical Graphics Corporation, St. Paul, MN). Machine calibration was performed prior to each session using a 3-liter syringe and calibration gases of known concentration of oxygen and carbon dioxide. VO_2_, minute ventilation, and RER were obtained continuously. Heart rate was measured during the last 15 s of each min. The maximal value for VO_2 _was taken as the average of the two highest consecutive values. To ensure that a true maximal VO_2 _had been attained, all of the following three criteria were met: subject failing to maintain treadmill elevation (% grade) for 15 consecutive seconds, an increase in VO_2 _of less than 100 ml· min^-1 ^despite an increase in workload, and a RER greater than 1.05. A best-fit linear regression equation, in which VO_2 _was plotted as a function of percent grade was developed for each subject. This equation was then used to determine the percent grade and subjects self-selected running velocity that corresponded to 70%VO_2_max for the subsequent endurance trials.

### Time to Exhaustion Test

Subjects exercised at the workload (velocity and % grade) that elicited 70% of their VO_2 _max on the treadmill. Exercise began 10 min following ingestion of the supplement or placebo. Machine calibration and subject preparation were performed as described above. During exercise VO_2 _and RER were measured continuously. Time to exhaustion was determined as the time that the subject could no longer maintain exercise intensity and/or reached volitional exhaustion.

### Questionnaires

Subjects were instructed to assess their subjective feelings of focus, energy and fatigue using a 10 cm visual analog scale (VAS). The VAS was assessed immediately before commencing exercise (PRE), following 10 min of exercise (EX10), and immediately post-exercise (IP). Subjects were asked to assess via a mark their feelings at that time with words anchored at each end of the VAS. Questions were structured as "My level of focus is:", with low and high serving as the verbal anchor representing the extreme ratings. Similarly, "My level of energy is:" was anchored with the verbal cues "low" and "high", while "My level of fatigue:" was anchored with the verbal cues "high" and "low". For fatigue, a higher score indicated less fatigue.

### Supplement

On each visit subjects ingested either the supplement or a placebo. The supplement is commercially marketed as 'Amino Impact™ ' (Koach, Sport and Nutrition, Langhorne, PA) and consisted of 26 g of a powder containing an energy matrix (2.05 g of caffeine, taurine, glucuronolactone), a proprietary amino acid matrix (7.9 g of L-leucine, L-isoleucine, L-valine, L-arginine and L-glutamine), 5 g of di-creatine citrate, and 2.5 g of β-alanine and mixed with 500 ml of water. The nutritional composition per serving of the supplement was 40 calories with 0 g of fat. The placebo consisted of 500 ml of water sweetened with 3 g of sucarlose (Splenda^®^, McNeil Nutritionals, Fort Washington, PA) and colored with red food coloring (McCormick Red Food coloring, McCormick & Company Hunt Valley, MD) to make it indistinguishable in appearance. The nutritional composition of the placebo contained no calories.

### Statistical Analyses

Performance data were analyzed using paired student's T-tests. Comparisons of subjects' measures of focus, energy and fatigue were accomplished using a repeated measures analysis of variance. In the event of a significant F-ratio, LSD post-hoc tests were used for pairwise comparisons. A criterion alpha level of p ≤ 0.05 was used to determine statistical significance. All data are reported as mean ± SD.

## Results

**Figure 1:**
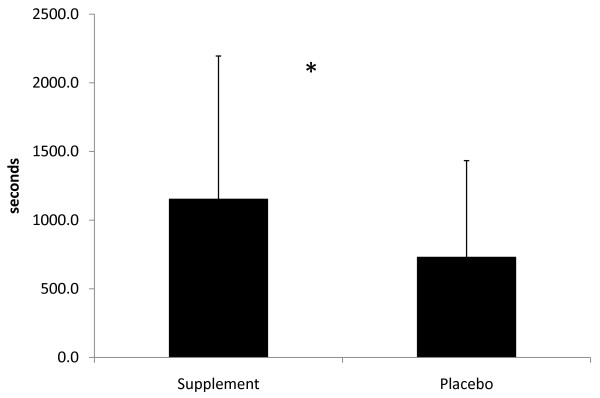
**Time to Exhaustion**. * = significant difference between groups.

Time to exhaustion was significantly greater (p = 0.012) during SUP than P (Figure 1). Subjects consuming the supplement were able to run 12.5% longer than when they consumed the placebo. Twelve of the 15 subjects were responders where improved performance during the SUP trial was observed. The magnitude of improvement in the responders ranged from 2.9 to 42.8%. The RER for all subjects was greater than 1.1 at the time of exhaustion. No significant difference in RER between trials was observed.

VAS scores of subjective measures of focus, energy and fatigue are presented in Table 1. Subjects consuming SUP reported significantly greater focus (p = 0.031), energy (p = 0.016), and less fatigue (p = 0.005) at PRE. Significant differences between groups were seen at EX10 for focus (p = 0.026) and energy (p = 0.004), but not fatigue (p = 0.123). No differences were seen at IP for either focus (p = 0.215), energy (p = 0.717) or fatigue (p = 0.430).

**Table 1:** Visual Analog Scale Scores for Subjective Measures of Focus, Energy and Fatigue.

		Focus	Energy	Fatigue
PRE	SUP	11.8 ± 1.9	11.7 ± 2.0	13.3 ± 2.8
	
	P	10.5 ± 2.8*	10.5 ± 2.8*	11.6 ± 2.9*

EX10	SUP	9.9 ± 3.1	9.7 ± 2.6	7.3 ± 3.4
	
	P	7.6 ± 3.7*	6.1 ± 3.1*	6.4 ± 3.3

IP	SUP	5.7 ± 5.0	3.2 ± 2.8	1.7 ± 1.8
	
	P	3.8 ± 3.6	2.8 ± 3.3	2.2 ± 2.2

* = Significant difference between groups

## Discussion

The results of this study indicate that an acute ingestion of the pre-exercise supplement Amino Impact™ containing caffeine, taurine, glucuronolactone, creatine, β-alanine, and the amino acids leucine, isoleucine, valine, glutamine and arginine can enhance time to exhaustion during moderate-intensity endurance exercise. In addition, consumption of this supplement 10-min prior to exercise appears to increase subjective feelings of focus, energy and reduce subjective feelings of fatigue before and during endurance exercise.

The most commonly used ingredient in energy drinks is caffeine. Caffeine has been shown to be an effective ergogenic agent by delaying fatigue and increasing time to exhaustion during endurance exercise [5-9]. This is thought to be related to caffeine's ability to enhance reliance on fat oxidation preserving muscle glycogen content [14]. Caffeine itself is only a mild central nervous system stimulator [15]. Therefore, additional ingredients (e.g., β-adrenergic receptor stimulators) are often combined with caffeine to increase the stimulatory response and provide additional ergogenic benefits. The synergistic effect of these ingredients has been demonstrated to increase subjective feelings of alertness, focus and energy [16-19], and improve reaction time to both auditory and visual stimuli [16]. Taurine is often combined with caffeine in energy drinks. Although its mechanism of action is not well understood, previous studies have shown that taurine by itself can improve endurance performance [20,21]. When combined with caffeine and glucuronolactone, ergogenic benefits of taurine have been confirmed in some investigations [12,13], but not others [22]. Differences between these studies may be related to the timing between ingestion and exercise (10 min versus 60 min between consumption and exercise in studies showing a positive response versus a negative response, respectively).

The use of BCAA in energy drinks is becoming more popular. Although BCAAs have been demonstrated to have an important role in protein synthesis [23], and enhance recovery from high-intensity exercise [24], several studies have suggested that BCAA may also improve cognition, focus and psychomotor function [25-28]. Egberts and colleagues [27] reported that BCAA supplementation can improve line tracing, steadiness, attention and auditory reaction time. Most studies demonstrating enhanced cognitive function from BCAA supplementation have been performed on subjects suffering from brain injury [25,26]. The mechanism underlying improved cognition has been suggested to be related to changes in amino acid concentrations within the brain [29]. During prolonged physical activity the use of BCAA may counteract or delay fatigue by decreasing the concentration of tryptophan and the synthesis of serotonin [28,30]. Serotonin has been implicated as a potential cause of central and mental fatigue during prolonged endurance activity [30], and decreases in this neurotransmitter may have an important role in minimizing or delaying performance decrements during fatiguing exercise. The results of this study suggest a contributory role of the BCAA towards delay in fatigue and enhanced focus. In addition, the combination of both arginine and BCAA has recently been shown to attenuate muscle proteolysis during endurance exercise [31].

The role that creatine may have had on the observed results is not clear. The ergogenic benefits of creatine supplementation have been well-documented [32]. These benefits have been expressed primarily during high intensity exercise and performance in strength/power events following approximately one week of supplementation. Creatine is generally not recognized as a potential ergogenic aid for endurance exercise. However, recent studies have focused on the role that phosphocreatine and the creatine kinase system play in mediating brain and neural function [33,34]. It is thought that 20% of the body's energy consumption may occur in the brain [33], thus an efficient ATP/PC replenishment system would be critical for normal brain function. Creatine is thought to provide important neuroprotection for the brain through enhancing energy metabolism in brain tissue, promoting antioxidant activities, improving cerebral vasculation (improved brain circulation) and acting as a brain cell osmolyte that can protect the brain against hyper-osmotic shock [35]. Creatine's neuroprotective properties also include stabilization of mitochondrial membranes, stimulation of glutamate uptake into synaptic vesicles and balance of intracellular calcium homeostasis [36]. These physiological roles for creatine suggest a potential neuroprotective effect that may become important during exhausting exercise. A recent study reported a 7-day loading dose of creatine improved cognitive function, enhanced psychomotor performance and improved mood state during a 36-hour sleep deprivation study [37]. Whether an acute dose of creatine can enhance subjective feelings of focus, energy and fatigue, as indicated by the results of this study, requires further investigation.

The additional ingredients found in Amino Impact™ include both glutamine and β-alanine. Glutamine is a non-essential amino acid that effectively modulates the immune response to exercise and possibly improves athletic performance by enhancing recovery and reducing muscle damage [38,39]. A recent investigation has suggested that glutamine may, in part, have an important role in enhancing fluid uptake during endurance exercise under dehydrated conditions [40]. However, its role in enhancing time to exhaustion where no notable hydration stress was present is unknown. It is possible that glutamine preserved hydration levels within the cell, but further research is warranted. Acute β-alanine supplementation has not been shown to have any role in enhancing endurance performance and likely had no effect in the observed results.

In conclusion, results of this study indicate that the supplement Amino Impact™ can significantly increase time to exhaustion during a moderate-intensity endurance run. In addition, ingestion of this supplement improved subjective feelings of focus, energy and fatigue at the onset and during the exercise protocol. These results provide evidence that the ingredients of this particular supplement, that have previously been shown to improve acute resistance training performance, can also benefit endurance exercise. This may have important implications as a pre-operation supplement for tactical athletes that are required to perform strength, power and endurance activities as part of their mission objectives.

## Competing interests

Supplement for this project was purchased through Inbounds Athletics. (Denver, CO). All researchers involved collected, analyzed, and interpreted the results from this study. JRH has a financial interest in Koach, Sport and Nutrition. No other author has financial interests concerning the outcome of this investigation. Publication of these findings should not be viewed as endorsement by the investigators, The College of New Jersey or the editorial board of the Journal of International Society of Sports Nutrition.

## Authors' contributions

ALW was the primary investigator, supervised all study recruitment, and data collection. AMG assisted with study recruitment and data collection. JK and NAR were co-authors, oversaw all aspects of study including recruitment, data/specimen analysis, and manuscript preparation. JRH was involved with study design, statistical analysis, and manuscript preparation. All authors have read and approved the final manuscript.

## References

[B1] Froiland K Koszewski W Hingst J Kopecky L Nutritional supplement use among college athletes and their sources of information Int J Sports Nutr Exerc Metab 2004 14 104 120 10.1123/ijsnem.14.1.104 15129934

[B2] Hoffman JR Faigenbaum AD Ratamess NA Ross R Kang J Tenenbaum G Nutritional supplementation and anabolic steroid use in adolescents Med Sci Sports and Exerc 2008 40 15 24 10.1249/mss.0b013e31815a5181 18091024

[B3] Desbrow B Leveritt M Awareness and use of caffeine by athletes competing at the 2005 Ironman Triathlon World Championships Int J Sport Nutr Exerc Metab 2006 16 545 558 17240785 10.1123/ijsnem.16.5.545

[B4] Petroczi A Naughton Dp Pearce G Bailey R Bloodworth A McNamee MJ Nutritional supplement use by elite young UK athletes: fallacies of advice regarding efficacy J Int Soc Sports Nutr 2008 5 22 10.1186/1550-2783-5-22 19077317 PMC2654424

[B5] Bruce CR Anderson ME Fraser SF Stepto NK Klein R Hopkins WG Hawley JA Enhancement of 2000-m rowing performance after caffeine ingestion Med Sci Sports Exerc 32 1958 1963 11079528 10.1097/00005768-200011000-00021

[B6] Graham TE Hibbert E Sathasivam P Metabolic and exercise endurance effects of coffee and caffeine ingestion J Appl Physiol 1998 85 883 889 9729561 10.1152/jappl.1998.85.3.883

[B7] Graham TE Spriet LL Performance and metabolic responses to a high caffeine dose during prolonged exercise J Appl Physiol 1995 78 867 874 1778925 10.1152/jappl.1991.71.6.2292

[B8] Hoffman JR Kang J Ratamess NA Jennings PF Mangine G Faigenbaum AD Effect of Nutritionally Enriched Coffee Consumption on Aerobic and Anaerobic Exercise Performance J Strength Cond Res 2007 21 456 459 10.1519/R-20326.1 17530975

[B9] Hogervorst E Bandelow S Schmitt J Jentjens R Oliveira M Allgrove J Carter T Gleeson M Caffeine improves physical and cognitive performance during exhaustive exercise Med Sci Sports Exerc 2008 40 1841 1851 10.1249/MSS.0b013e31817bb8b7 18799996

[B10] Kalmar JM The influence of caffeine on voluntary muscle activation Med Sci Sports Exerc 2005 37 2113 2119 10.1249/01.mss.0000178219.18086.9e 16331138

[B11] Woolf K Bidwell WK Carlson AG Effect of caffeine as an ergogenic aid during anaerobic exercise performance in caffeine naïve collegiate football players J Strength Cond Res 2009 1363 1369 19620930 10.1519/JSC.0b013e3181b3393b

[B12] Hoffman JR Ratamess NA Ross R Shanklin M Kang J Faigenbaum AD Effect of a Pre-Exercise 'High-Energy' Supplement Drink on the Acute Hormonal Response to Resistance Exercise J Strength Cond Res 2008 22 874 882 18438227 10.1519/JSC.0b013e31816d5db6

[B13] Ratamess NA Hoffman JR Ross R Shanklin M Faigenbaum AD Kang J Effects of an Amino Acid/Creatine/Energy Supplement on Performance and the Acute Hormonal Response to Resistance Exercise Int J Sport Nutr Exerc Metab 2007 17 608 623 18156665 10.1123/ijsnem.17.6.608

[B14] Spriet LL Caffeine and performance Int J Sport Nutr 1995 5 S84 S99 7550260 10.1123/ijsn.5.s1.s84

[B15] Sawynok J Pharmacological rationale for the clinical use of caffeine Drugs 1995 49 37 51 10.2165/00003495-199549010-00004 7705215

[B16] Hoffman JR Kang J Ratamess NA Hoffman MW Tranchina CP Faigenbaum AD Examination of a high energy, pre-exercise supplement on exercise performance J Int Soc Sports Nutr 2009 6 2 10.1186/1550-2783-6-2 19126213 PMC2621122

[B17] Scholey AB Kennedy DO Cognitive and physiological effects of an "energy drink": an evaluation of the whole drink and of glucose, caffeine, and herbal flavouring fractions Psychopharm 2004 176 320 330 10.1007/s00213-004-1935-2 15549275

[B18] Smit HJ Cotton JR Hughes SC Rogers PJ Mood and cognitive performance effects of "energy" drink constituents: caffeine, glucose and carbonation Nutr Neurosci 2004 7 127 139 10.1080/10284150400003041 15526987

[B19] Smith A Effects of caffeine on human behavior Food Chem Toxicol 2002 40 1243 1255 10.1016/S0278-6915(02)00096-0 12204388

[B20] Miyazaki T Matsuzaki Y Ikegami T Miyakawa S Doy M Tanaka N Bouscarel B Optimal and effective oral doses of taurine to prolong exercise performance in rat Amino Acids 2004 27 291 298 10.1007/s00726-004-0133-1 15503230

[B21] Zhang M Izuma I Kagamimori S Sokejima S Yamagami T Liu Z Qi B Role of taurine supplementation to prevent exercise-induced oxidative stress in healthy young men Amino Acids 2004 26 203 207 10.1007/s00726-003-0002-3 15042451

[B22] Candow DG Kleisinger AK Grenier S Dorsch KD Effect of sugar-free red bull energy drink on high-intensity run time-to-exhaustion in young adults J Strength Cond Res 2009 23 1271 1275 19528841 10.1519/JSC.0b013e3181a026c2

[B23] Tipton KD Rasmussen BB Miller SL Wolf SE Owens-Stovall SK Petrini BE Wolfe RR Timing of amino acid-carbohydrate ingestion alters anabolic response of muscle to resistance exercise Am J Phys Endocr Metab 2001 281 E197 E206 10.1152/ajpendo.2001.281.2.E197 11440894

[B24] Hoffman JR Ratamess NA Tranchina CP Rashti SL Kang J Faigenbaum AD Effect of Protein Ingestion on Recovery Indices Following a Resistance Training Protocol in Strength/Power Athletes Amino Acids 2009 in press 19347247 10.1007/s00726-009-0283-2

[B25] Aquilani R Iadarola P Contardi A Boselli M Verri M Pastoris O Boschi F Arcidiaco P Viglio S Branched-chain amino acids enhance the cognitive recovery of patients with severe traumatic brain injury Arch Phys Med Rehab 2005 86 1729 1735 10.1016/j.apmr.2005.03.022 16181934

[B26] Cole JT Mitala CM Kundu S Verma A Elkind JA Nissim I Cohen AS Dietary branched chain amino acids ameliorate injury-induced cognitive impairment Proc Natl Acad Sci 2010 107 366 371 10.1073/pnas.0910280107 19995960 PMC2806733

[B27] Egberts EH Schomerus H Hamster W Jurgens P Branched chain amino acids in the treatment of latent portosystemic encephalopathy. A double blind placebo controlled crossover study Gastroenterology 1985 88 887 895 3882509 10.1016/s0016-5085(85)80004-4

[B28] Fernstrom JD Branched-chain amino acids and brain function J Nutr 2005 135 1539s 1546s 15930466 10.1093/jn/135.6.1539S

[B29] Meeusen R Watson P Amino acids and the brain: do they play a role in "central fatigue"? Int J Sports Nutr Exerc Metab 2007 17 S37 S46 10.1123/ijsnem.17.s1.s37 18577773

[B30] Davis JM Alderson NL Welsh RS Serotonin and central nervous system fatigue: nutritional considerations Am J Clin Nutr 2000 72 573S 578S 10919962 10.1093/ajcn/72.2.573S

[B31] Matsumoto K Mizuno M Mizuno T Dilling-Hansen B Lahoz A Bertelsen V Münster H Jordening H Hamada K Doi T Branched-chain amino acids and arginine supplementation attenuates skeletal muscle proteolysis induced by moderate exercise in young individuals Int J Sports Med 2007 28 531 538 10.1055/s-2007-964940 17497593

[B32] Hoffman JR Stout JR Earle RW, Baechle TR Performance-Enhancing Substances Essentials of Strength and Conditioning 2008 3 Human Kinetics: Champaign, IL 179 200

[B33] Shulman RG Rothman DL Behar KL Hyder F Energetic basis of brain activity: implications for neuroimaging Trends Neurosci 2004 27 489 495 10.1016/j.tins.2004.06.005 15271497

[B34] Stockler S Schutz PW Salomons GS Cerebral creatine deficiency syndromes: clinical aspects, treatment and pathophysiology Subcell Biochem 2007 46 149 66 full_text 18652076 10.1007/978-1-4020-6486-9_8

[B35] Andres RH Ducray AD Schlattner U Wallimann T Widmer HR Functions and effects of creatine in the central nervous system Brain Res Bul 2008 76 329 343 10.1016/j.brainresbull.2008.02.035 18502307

[B36] Ellis AC Rosenfeld J The role of creatine in the management of amyotrophic lateral sclerosis and other neurodegenerative disorders CNS Drugs 2004 18 967 980 10.2165/00023210-200418140-00002 15584767

[B37] McMorris T Harris RC Howard AN Langridge G Hall B Corbett J Dicks M Hodgson C Creatine supplementation, sleep deprivation, cortisol, melatonin and behavior Physiol Behav 2007 90 21 28 10.1016/j.physbeh.2006.08.024 17046034

[B38] Castell LM Newsholme EA Glutamine and the effects of exhaustive exercise upon the immune response Can J Physiol Pharmacol 1998 76 524 532 10.1139/cjpp-76-5-524 9839078

[B39] Favano A Santos-Silva PR Nakano EY Pedrinelli A Hernandez AJ Greve JM Peptide glutamine supplementation for tolerance of intermittent exercise in soccer players Clinics 2008 63 27 32 10.1590/S1807-59322008000100006 18297203 PMC2664173

[B40] Hoffman JR Ratamess NA Kang J Rashti SL Kelly N Gonzalez AM Stec M Andersen S Bailey BL Yamamoto LM Hom LL Kupchak BR Faigenbaum AD Maresh CM Examination of the efficacy of acute L-Alanyl-L-Glutamine during Hydration Stress in Endurance Exercise J Int Soc Sports Nutr 2010 7 8 10.1186/1550-2783-7-8 20181080 PMC2851582

